# A novel TEM grid sampler for airborne particles to measure the cell culture surface dose

**DOI:** 10.1038/s41598-020-65427-w

**Published:** 2020-05-21

**Authors:** Sonja Mülhopt, Christoph Schlager, Markus Berger, Sivakumar Murugadoss, Peter H. Hoet, Tobias Krebs, Hanns-Rudolf Paur, Dieter Stapf

**Affiliations:** 1 Karlsruhe Institute of Technology (KIT), Institute for Technical Chemistry, Eggenstein-Leopoldshafen, 76344 Germany; 2 Vitrocell Systems GmbH, Waldkirch, 79183 Germany; 3 KU Leuven, Environment and Health, Leuven, 3000 Belgium

**Keywords:** Nanotoxicology, Cell-particle interactions

## Abstract

The applied surface dose is a key parameter for the measurement of toxic effects of airborne particles by air liquid interface exposure of human lung cells. Besides online measurement of the deposited particle mass by quartz crystal microbalance frequently other dose metrics such as particle size distribution, surface and agglomeration state are required. These particle properties and their spatial distribution can be determined by digital processing of micrographs obtained by transmission electron microscopy (TEM). Here, we report the development and characterization of a novel holder for film coated TEM copper grids, which allows for sampling under identical geometric and ambient conditions as in a cell culture chamber. The sample holder avoids artefacts by reliable grounding of the grids and improves handling of the grids to prevent damage of the sensitive film. This sample holder is applied during exposure experiments with titanium dioxide nanoparticles. The measured dose of 0.2 µg/cm² corresponds well to the mass loading signal of the quartz crystal microbalance. Additionally, the spatial distribution of particles on the sampling surface shows a good homogeneity of deposition. This novel sampling method allows verifying other dosimetry methods and gives additional information about particle properties and homogeneity of the dose.

## Introduction

The applied surface dose of particles per area is an essential parameter to assess the inhalation toxicity of aerosols at the air-liquid interface (ALI)^1^. Due to the low gas velocities, only a small fraction of the airborne particles deposits on the cells^2–4^. As particle size and agglomeration state play an important role for the deposition behavior^5,6^, digital image processing of electron micrographs delivers useful data regarding the surface dose of particles on exposed cells. Image evaluation of transmission electron microscopy (TEM) samples is a highly sensitive method to determine deposition pattern and spatial distribution of nanoparticles. To obtain these important data, film coated copper grids should be exposed under identical geometric conditions as the cells, but when placing TEM grids on the cell culture membrane they often get lost or, when being fixed, the film is destroyed. Therefore, we developed a TEM grid sampler^7^, which allows for sampling under relevant air-liquid interface conditions as in a cell culture chamber. It was applied for exposure studies with titanium dioxide nanoparticles. The surface dose and the spatial distribution on the membrane delivers important data for measuring dose-response relationships in toxicity studies.

## Methods

### Air-Liquid Interface (ALI) exposure of biological surfaces

The prevailing method of studying the health effects of aerosols *in-vitro* bases on submerged exposure of collected particulate matter, suspended in culture medium. However, this method neglects the gas phase including their interactions with particles and cells. It does not represent the actual process in the human lung and may change the properties of the investigated particles. Suspending particles in the medium may affect the agglomeration state and particle size due to solubility or protein corona. Especially the particle surface interactions with proteins can influence the biological responses as shown by several authors^8,9^. Due to these disadvantages, expert panels recommend the exposure at the ALI^10–12^. The cell cultures are exposed to a particle loaded airflow without artefacts due to medium covering the cell surface. To simulate physiological conditions the cell layer is seeded on a membrane insert and supplied with medium from the lower compartment while the aerosol flow, temperature, and humidity are adjusted to the conditions resembling the lower human lung region. To achieve reproducible conditions, a fully automated ALI exposure station was developed^3,13^. The exposure station offers a complete measurement system for parallel exposure of up to 24 human lung cell cultures towards gases, nanoparticles and complex mixtures such as combustion aerosols. An internal negative control using humidified synthetic air is also implemented and the particle dose per time can be increased by electrostatic particle deposition. Applications of the ALI exposure station are environmental atmospheres and technical emission sources like ship diesel exhaust as reported by Oeder *et al*.^14^ and Sapcariu *et al*.^15^, as well as biomass combustion sources^16^.

### TEM grid sampler

**Figure 1:**
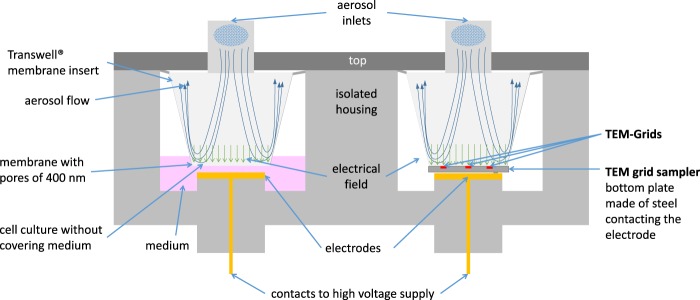
Comparison of the exposure scenarios for cell cultures in a Transwell membrane insert (left) and the TEM grid sampler (right).

We constructed a sampling tool, which was manufactured by Vitrocell Systems (Waldkirch, Germany) for exposing the TEM grids that reflects the exposure scenario of the cells cultured on the membrane of a Transwell membrane insert. Cell cultures are seeded on the porous polycarbonate membrane, which is contacted to the medium below the Transwell membrane insert (Fig. 1, left). For increasing the dose, which corresponds to the deposited particle mass or number, the medium and cell culture, can be contacted to a high electric potential forming an electrical field between aerosol inlet and cell culture. This forces charged particles of the opposite polarity to the membrane and increases the fraction of depositing particles by the factor up to 10^3^ ^17^.

In consequence, the sampler was developed to achieve the following goals (Fig. 1 right):

- Identical geometry in the aerosol deposition region of TEM grid sampler and Transwell membrane insert resulting in identical deposition pattern
- Electroconductive surface in the sampler bottom
- Electrical contact to the electrode providing enhanced particle deposition
- Non-conductive walls with contact to the top and the housing, both grounded

The development was performed based on a 6-well Transwell membrane insert as it is our standard for ALI cell cultivation, but also a 12-well insert is already realized and similar formats will be possible. The TEM grid sampler can be used in most ALI system that uses membrane inserts of the 6- or 12-well format e.g. Vitrocell Cloud^18^, Cultex^19^, NACIVT^20^ or PRIT^21^. The inner geometry is identical to the membrane insert but the outer dimension is reaching into the space, which usually is filled with medium.

**Figure 2:**
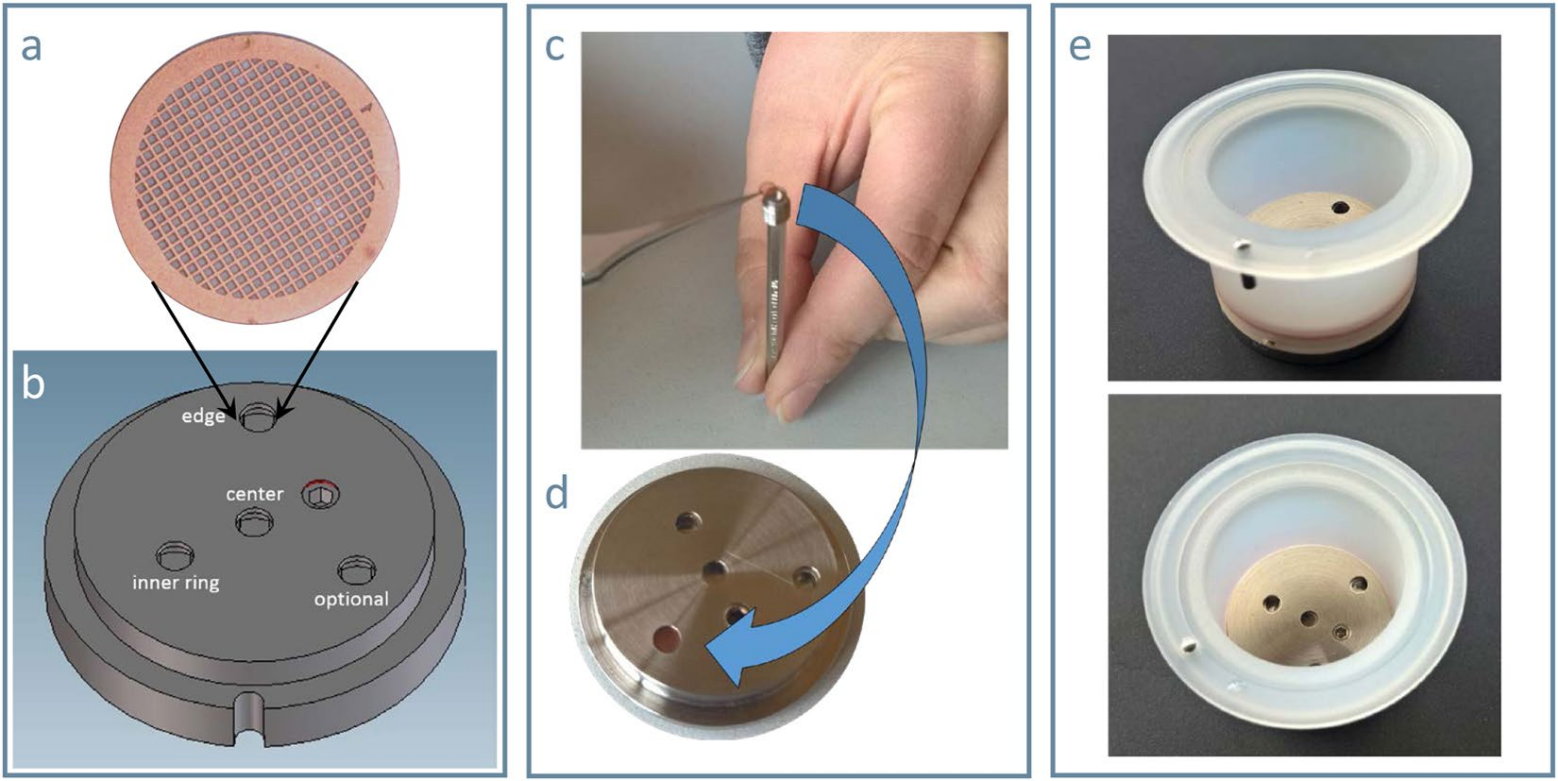
TEM grid sampler: (**a**) micrograph of a Formvar film coated copper grid for TEM with 200 mesh (**b**) scheme of base plate with different positions. (**c**) inserting a TEM grid into the grid holder (**d**) fixed grid holder in the base plate. € Complete TEM grid sampler with housing simulating the membrane inserts for cell culturing and exposure.

In detail, the TEM grid sampler consists of two parts: the base plate made of stainless steel, with four wells, which accommodate grid holders (Fig. 2a,c). Up to four grids can be installed in the base plate, representing different positions on the radius of the base plate, more specific the centre, the edge and in-between (Fig. 2b,d). The base plate is fixed as bottom in the housing, to mimic the geometry of a Transwell membrane insert (Fig. 2e).

### Aerosol generation, exposure, and measurement

The TiO_2_ nanoparticles used in this study are JRC representative test material JRCNM10200a and similar to JRC representative test material NM100. Detailed information on synthesis and physico-chemical properties of NM100 is provided in the JRC report^22^. Using TEM, the primary size of the spherical shaped TiO_2_ particle is determined as 117 nm (Feret minimum). Pristine (bare) TiO_2_ nanoparticles are used and well dispersed in stock solution according Murugadoss *et al*.^23^. It was confirmed that the TiO_2_ nanoparticles predominantly exist as single particles or very small agglomerates. A suspension of 1 g/l TiO_2_ NP in ultrapure water was sonicated for 30 min and aerosolized using a two phase nozzle with a drying zone of silicagel according sheet 2 of VDI guideline 3491^24^. During aerosolisation, the suspension was stirred permanently.

1 m³/h of dried TiO_2_ NP aerosol enters the ALI exposure system^3,13^ where a typical exposure experiment was performed: the TEM grid sampler, equipped with three Formvar film coated copper grids with 200 mesh, type SF162 (Plano GmbH, Wetzlar, Germany) was exposed towards an aerosol flow of 100 ml/min for 4 hours. Four TEM grid samplers were installed in a Vitrocell exposure station and exposed in parallel to the aerosol flow at 0, 400, 800 and 1200 Volt.

The particle number size distribution was measured directly in the conditioning reactor of the ALI exposure station using a scanning mobility particle sizer U-SMPS (Palas GmbH, Karlsruhe, Germany). The U-SMPS operates continuously during the 4-hour experiment measuring the number size distribution every 5 min. The number concentration in every channel was corrected regarding diffusion losses^25,26^ and averaged. The resulting particle size distribution was fitted by a log-normal distribution to determine the modal value x_m_, geometric standard deviation σ_g_ and the total number concentration N of the aerosol.

The deposited dose without electrostatic deposition was monitored online using the quartz crystal microbalance QCM^27^.

### Image evaluation

From each grid, 10 micrographs were taken by TEM EM 109 (Carl Zeiss Microscopy GmbH, Oberkochen, Germany) at a magnification of 4000. These micrographs were processed regarding particle load and particle size using the software ImageJ^28^. The particle number per area (1/cm²) was determined by creating a binary image of the micrograph and applying watershed segmentation, followed by particle analysis. The particle diameter was determined as area equivalent diameter. The particle mass per area was calculated from the deposited particle number and diameter using the effective particle density of 1.86 g/cm³ as measured by the volumetric method^29,30^. The results are expressed as means + standard deviation (SD) of several independent experiments as indicated in the legends and graphs, respectively. The significance of difference between two mean values was assessed by Student’s t test. A p value < 0.05 was considered to be statistically significant.

## Results and discussion

**Figure 3:**
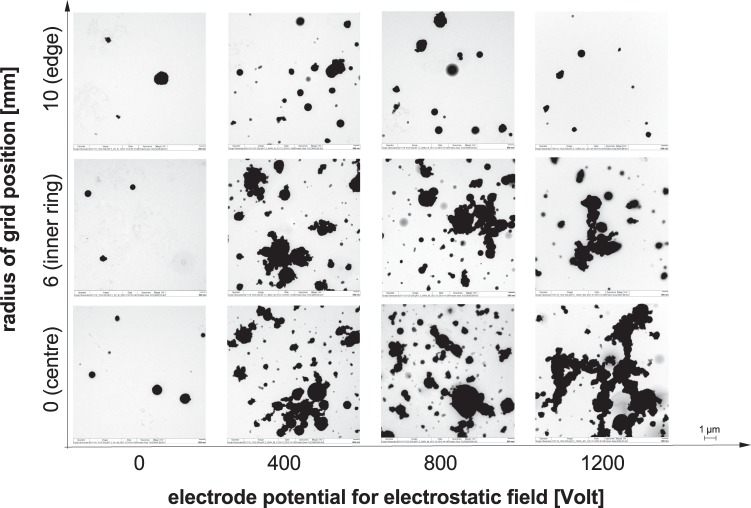
TEM images of exposed copper grids: Magnitude 4000, Voltage 80 kV; Representative image of the grids in the different positions of the grid sampling tool, from bottom to top: centre, inner ring, edge. Exposure experiments performed with increasing electrostatic deposition, starting without electric potential on the grid holding steel plate on the left going up to 1200 V on the right.

The images of the particle loaded TEM grids show particles, which are partially agglomerated and partially deposited as primary particles. No significant difference between the centre and the inner area could be observed, however the concentration of particles per area was significantly lower at the edge of the deposition surface (Fig. 3) due to the electrical field which is limited to the area covered by the aerosol inlet^17^. For the highest electrical field in combination with reaching saturation for the charged fraction they deposit fast in the centre and the decrease towards the edge increases. Increasing the electrostatic potential increases the deposited particle fraction.

**Figure 4:**
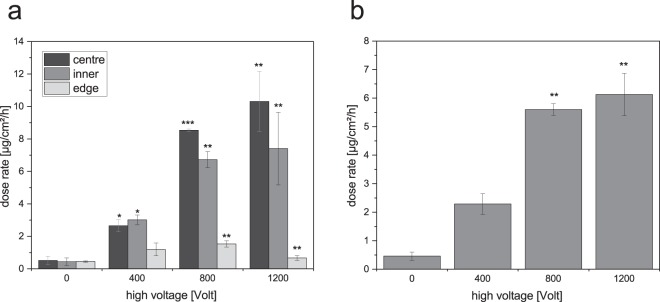
Dose rates in µg/cm²/h in three positions of the TEM grid sampler exposed to TiO2 aerosol deposited without and with electrical fields. a: influence of the position and applied voltage on the dose rate b: mean TiO_2_ masses deposited on the membrane surface per hour depending on the and applied voltage in the range of zero, 400, 800 and 1200 V. The values are means ± standard deviation obtained from three (two for 400 volts) independent experiments, *p < 0.05, **p < 0.01, ***p < 0.001 in comparison to no electrostatic deposition.

Ten micrographs of each grid were processed using ImageJ to measure the mean particle diameter d_P_ and the particle number per area. Dose rates were determined in dependence on location of the grid and electrostatic potential (Fig. 4a). Particle deposition increases with increasing the electrostatic potential (Fig. 4a,b). When reaching the highest potential of 1200 Volt the increase becomes less significant which indicates that all available charged particles were deposited. Due to limitation in the high voltage supply, higher voltages could not be tested to verify this assumed limit.

On the TEM images (Fig. 3) the typical deposition pattern^17,31^ was observed, but especially for the dose without electrostatic deposition low numbers of particles were detected and counted. This causes higher standard deviations as well as in the case of the highest electrostatic deposition causing bigger agglomerates. According to Fig. 3 particles were less agglomerated without an electrostatic field. However, agglomerates were observed when an electrostatic field is applied. Further investigations using the novel TEM grid sampler and numerical simulation are required to clarify the mechanism of agglomeration. Overall standard deviations in the range of 4 to 31% for the mean of the three independent experiments show that exposures of TEM grids towards aerosol could be performed reproducibly.

**Figure 5:**
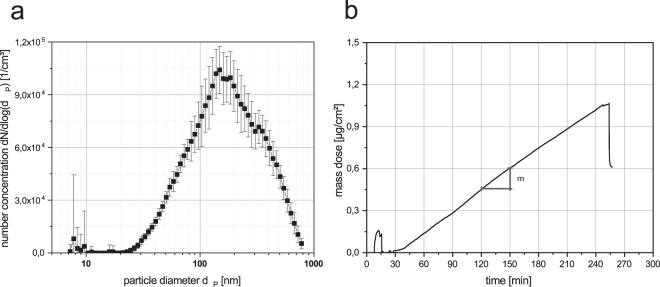
Aerosol characteristics and online dose measurement (**a**) particle number size distribution determined by SMPS; (**b**) QCM signal during the exposure. The slope m in the range between 30 to 240 min was used to calculate the dose rate.

The particle size distribution measured by U-SMPS shows a lognormal distribution (Fig. 5a) with a modal value of x_m_ = 160 nm, a geometric standard deviation of σ_g_ = 1.95 and a total number concentration of N = 1.68E + 05 1/cm³. Using the particle size distribution data and the effective density the aerosol mass concentration is estimated to c_M_ = 2.11 mg/m³.

The particle mass diffusional deposited on the surface was monitored online by QCM (Fig. 5b). The dose rate was determined from the slope of the particle loading = 4.9 ng/cm²/min. From this the 4 hours dose was calculated to 1.17 µg/cm².

The recalculation of the size distribution obtained by digital processing of TEM micrographs provides a surface dose of 1.27 µg/cm², which is only 8% higher than the QCM measurement.

Comparison of the surface dose estimated by two independent methods results in remarkable agreement. However, considering the assumptions during processing and recalculation of TEM data and the uncertainty of the density of agglomerated particles we assume that the mass measurement by QCM provides still the best estimate of the surface dose, since it requires no information on particle density.

## Conclusions

This report presents a novel dosimetry tool for evaluating the particle deposition in ALI exposure chambers for cell culture inserts as Transwell membrane inserts. With this sampling tool for the exposure of TEM grids dosimetry experiments were performed with the following advantages:

- The method provides a highly sensitive surface dose measurement. Due to the possibility to analyse even single particles on the surface area of a grid this method enables to detect extremely low concentrations of deposited nanoparticles.
- The TEM grids are fixed in a reproducible way and at defined positions. The grid holder allows to fix the grids in the deposition surface without use of glue or other additional chemicals.
- The stainless steel plate can be contacted to the electrode and so be used for electrostatically enhanced deposition. By assuring a conductive contact between electrode and deposition surface including the TEM grids the investigation of electrostatic deposition is possible, showing e.g. the increase factors as a function of high voltage.
- Reproducible investigation of the spatial particle distribution on the deposition surface is possible.

We showed that the TEM grid sampler delivers reproducibly exposed TEM grids for taking high quality images for further image evaluation. A comparison of this dosimetry data shows similar results as the established QCM method. Furthermore, it can be used with different electrostatic potentials to determine the increase factors of dose.

## Data Availability

The datasets generated during and analysed during the current study are available from the corresponding author upon reasonable request.
